# Haemodynamic efficacy of microaxial left ventricular assist device in cardiogenic shock: a systematic review and meta-analysis

**DOI:** 10.1007/s12471-019-01351-7

**Published:** 2019-12-06

**Authors:** D. I. M. van Dort, K. R. A. H. Peij, O. C. Manintveld, S. E. Hoeks, W. J. Morshuis, N. van Royen, T. Ten Cate, G. S. C Geuzebroek

**Affiliations:** 1 Department of Cardiothoracic Surgery, Radboud University Medical Centre, Nijmegen, The Netherlands; 2 Department of Cardiology, Thoraxcenter, Erasmus MC, Rotterdam, The Netherlands; 3 Department of Anaesthesiology, Erasmus MC, Rotterdam, The Netherlands; 4 Department of Cardiology, Radboud University Medical Centre, Nijmegen, The Netherlands

**Keywords:** Impella, Haemodynamic monitoring, Cardiogenic shock, Heart failure, Left ventricular assist device

## Abstract

The Impella percutaneous mechanical circulatory support device is designed to augment cardiac output and reduce left ventricular wall stress and aims to improve survival in cases of cardiogenic shock. In this meta-analysis we investigated the haemodynamic effects of the Impella device in a clinical setting. We systematically searched all articles in PubMed/Medline and Embase up to July 2019. The primary outcomes were cardiac power (CP) and cardiac power index (CPI). Survival rates and other haemodynamic data were included as secondary outcomes. For the critical appraisal, we used a modified version of the U.S. Department of Health and Human Services quality assessment form. The systematic review included 12 studies with a total of 596 patients. In 258 patients the CP and/or CPI could be extracted. Our meta-analysis showed an increase of 0.39 W [95% confidence interval (CI): 0.24, 0.54], (*p* = 0.01) and 0.22 W/m^2^ (95% CI: 0.18, 0.26), (*p* < 0.01) for the CP and CPI, respectively. The overall survival rate was 56% (95% CI: 0.50, 0.62), (*p* = 0.09). The quality of the studies was moderate, mostly due to the presence of confounders. Our study suggests that in patients with cardiogenic shock, Impella support seems effective in augmenting CP(I). This study merely investigates the haemodynamic effectiveness of the Impella device and does not reflect the complete clinical impact for the patient.

**Electronic supplementary material:**

The online version of this article (10.1007/s12471-019-01351-7) contains supplementary material, which is available to authorized users.

## Introduction

The Impella (Abiomed, Danvers, MA, USA) is a percutaneous mechanical circulatory support (MCS) device consisting of a non-pulsatile microaxial flow pump based on the Archimedes screw principle that propels blood from the left ventricle into the ascending aorta. Aside from increasing blood flow, the Impella device aims to reduce ventricular wall stress, thereby unloading the left ventricle, reducing oxygen consumption and decreasing infarct size [1]. A series of Impella devices are available for left ventricular support. The Impella 2.5 and Impella CP can provide haemodynamic support up to 2.5 and 3.7 l/min, respectively. The strongest Impella, the Impella 5.0, can deliver up to 5 l/min of haemodynamic support. However, this includes the use of a 21 Fr pump motor, making the implantation in the acute setting more challenging [2, 3].

MCS devices have been increasingly used as a key element in the management of patients with cardiogenic shock (CS) [4]. Based on the results of the USpella Registry, which showed a significant increase of cardiac output (CO) [5], the Impella received FDA approval in 2016 for the treatment of CS. Increased flow is beneficial in CS, since low CO and reduced perfusion pressure are the bases of CS syndrome [6]. However, these two factors are intertwined, and a decreased output does not necessarily indicate a decreased perfusion pressure and vice versa. The product of these combined parameters is the cardiac power (CP), and is the strongest haemodynamic predictor of mortality in the SHOCK trial registry [7]. This finding was confirmed in a more recent study, where the cardiac power index (CPI) was found to be the best haemodynamic predictor of survival in a CS population [8].

As CP and CPI are the best predictors for survival, we focused specifically on the effects of Impella on CP(I).

### Methods

We performed a systematic review and meta-analysis on the haemodynamic effects of the Impella during CS. Survival rate was a secondary outcome.

#### Search strategy

Medical literature databases PubMed/Medline and Embase were searched using the following keywords: (((Impella[tiab] OR (microaxial[tiab] OR axial[tiab]) AND flow[tiab] AND (pump*[tiab] OR catheter*[tiab]) OR percutaneous left ventricular assist device*[tiab])) AND (((cardiogenic shock[tiab] OR cardiac shock[tiab] OR cardiovascular shock[tiab] OR heart shock[tiab] OR acute cardiac failure[tiab] OR acute decompensated heart failure[tiab] OR acute heart insufficiency[tiab] OR acutely decompensated heart failure[tiab] OR ADHF[tiab] OR forward heart failure[tiab] OR low cardiac output[tiab] OR low output syndrome[tiab] OR systolic dysfunction[tiab])) OR (((Shock, Cardiogenic[Mesh] OR Heart Failure[Mesh:noexp] OR Heart Failure, Systolic[Mesh])) OR “Myocardial Infarction”[Mesh]))). A methodological filter was used to limit the results to adult humans. The search was last updated on 9 July 2019.

#### Inclusion and exclusion criteria

This article is in accordance with the PRISMA guidelines (see Electronic Supplementary Material for checklist, online Table 1) [9]. Studies eligible for inclusion were original articles that met the following criteria: retrospective, prospective cohort studies and randomised controlled trials in CS patients, with a reported CS. We excluded letters, case reports and studies that focused on high-risk percutaneous coronary intervention (PCI). No further restrictions on publication date, status or language were imposed.

The search was then loaded into Endnote X8 and possible duplicates were deleted. The two reviewers independently reviewed all titles, abstracts and manuscripts to determine whether they met the inclusion criteria. Disagreement between reviewers (K.P. and D.D.) was resolved by consensus. Reference lists from eligible studies were checked to identify additional studies and citations. For the critical appraisal, we used an adapted version of the U.S. Department of Health and Human Services quality assessment form (see Fig. 6; [10]).

Both reviewers independently extracted the data from all the selected manuscripts. For haemodynamic parameters the CO, cardiac index (CI), mean arterial pressure (MAP), CP, CPI and pulmonary wedge pressure (PWP) were obtained. For non-haemodynamic parameters, type and duration of MCS, mechanical ventilation, cardiopulmonary resuscitation, gender and survival were also extracted from the individual studies.

#### Outcomes

Primary outcomes were CP and CPI. The CP is calculated as: CO × MAP/451 [7]. The CPI was computed by substituting CO with CI in the respective formula.

Secondary outcomes included survival, type and duration of MCS, mechanical ventilation, cardiopulmonary resuscitation, gender and other haemodynamic data (CO, CI, MAP, PWP).

#### Statistical analysis

All data were analysed using Review Manager 5.3.5 and Rstudio. Categorical variables were presented as percentages. Continuous variables were presented as range or mean ± standard deviation (SD). For continuous variables reported as median ± interquartile range, the mean and SD were estimated by using the formula as proposed by Hozo et al. [11]. Not all studies mentioned the CP or CPI directly; therefore the missing CP or CPI and accessory SD were calculated according to the appropriate formulas [11].

Heterogeneity defined as variation among the results of the individual studies was assessed with Cochrane’s Q‑statistic (*p*_chance_ and *I*^2^ statistic). Random effects models were used to calculate mean pooled differences of haemodynamic data between baseline and Impella support for CP and CPI. A subgroup analysis of the Impella 2.5 and 5.0 was made. For survival rates, the overall proportion from studies reporting a single proportion was calculated. Note that since not all variables were measured in all patients and all studies, the number of patients and studies per meta-analysis is different.

### Results

#### Study characteristics

**Fig. 1:**
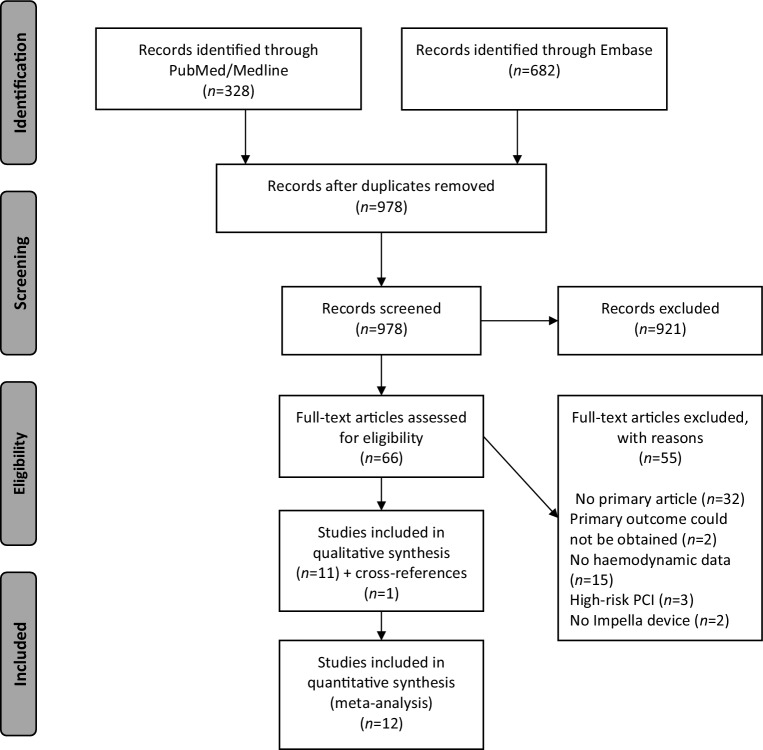
Flow diagram of the included studies [9]. *PCI* Percutaneous coronary intervention

Our systematic literature search in PubMed/Medline and Embase resulted in 946 records (Fig. 1). After exclusion, 12 articles (including 1 via cross-reference) remained for qualitative and quantitative synthesis and meta-analysis [5, 12–22]. Nine of the 12 studies were observational. Two were prospective single-arm trials and one study was a randomised controlled trial. The Impella 2.5 was investigated in five studies, the Impella 5.0 in six studies and one study investigated both devices.

#### Patients

The systematic review included 12 studies with a total of 596 patients studied. Patient characteristics are shown in Tab. 1.

**Table 1:** Study characteristics included in the meta-analysis

Study	Year	Study type	Number of patients	Type of Impella	Indication	CS-AMI	Age (years)	Male (%)	MV (%)	CPR (%)	Support (days)	LVEF (%)
*Meyns *[12]	2003	Registry	13	5.0	CS	6/16	60 ± n.a.	69	–	–	4.0 ± n.a.	–
*Dens *[13]	2006	Prospective	11	2.5	CS-AMI	11	61 ± 11	73	–	–	0.9 ± 0.7	29 ± 11
*Seyfarth *[14]	2008	RCT	13	2.5	CS-AMI	13	65 ± 10	62	92	85	0.9 ± 0.8	27 ± n.a.
*Bresson *[15]	2011	Registry	5 (9)	5.0	CS	6/11	50 ± 14	83	100	–	12 ± 7.3	–
*Griffith *[16]	2013	Prospective	16	5.0	CS*	0/16	58 ± 9	81	–	–	3.7 ± 2.9	23 ± 7
*O’Neill *[5]	2014	Registry	23 (154)	2.5	CS-AMI	154	64 ± 13	71	66	49	1.2 ± 1.9	26 ± 13
*Casassus *[17]	2015	Registry	9 (22)	2.5	CS-AMI	22	58 ± 12	59	55	55	1.5 ± 1.1	27 ± 8
*Lima *[18]	2016	Registry	21 (40)	5.0	ESHF	0/40	55 ± 13	78	65	–	7 ± 5	12 ± 5
*Schiller *[19]	2016	Registry	66	2.5/5.0	CS	26/66	55 ± 2	65	–	–	7.4 ± 0.8	28 ± 14
*Joseph *[20]	2016	Registry	35 (180)	2.5	CS-AMI	180	66 ± 13	73	77	55	n.a.	26 ± 12
*Mastroianni *[21]	2017	Registry	14	5.0	CS*	0/14	64 ± 15	79	71	–	8.5 ± 4.7	–
*Hall *[22]	2018	Observational	58	5.0	ESHF	0/58	55 ± 13	79	24	–	7 ± 5	13 ± 7

*CS-AMI* cardiogenic shock complicating acute myocardial infarction; CS* cardiogenic shock post-surgery; *ESHF* end-stage heart failure; *MV* mechanical ventilation; *CPR* cardiopulmonary resuscitation; *LVEF* left ventricular ejection fraction; – not available

Indications for Impella implant were CS after acute myocardial infarction (CS-AMI) in 380 (64%), end-stage heart failure in 96 (16%) and post-cardiac surgery in 30 patients (5%). The remaining 88 (15%) patients had Impella implanted for various causes of CS.

During hospital admission, 55–100% of the patients received mechanical ventilation and 49–85% had cardiopulmonary resuscitation prior to Impella implantation. In all studies, patients were pharmacologically supported by inotropic and/or vasopressor agents and in 9 of the 12 studies (74% of all patients) PCI was conducted. The mean duration of support with the Impella was 0.9–12 days.

#### Meta-analysis

**Fig. 2:**
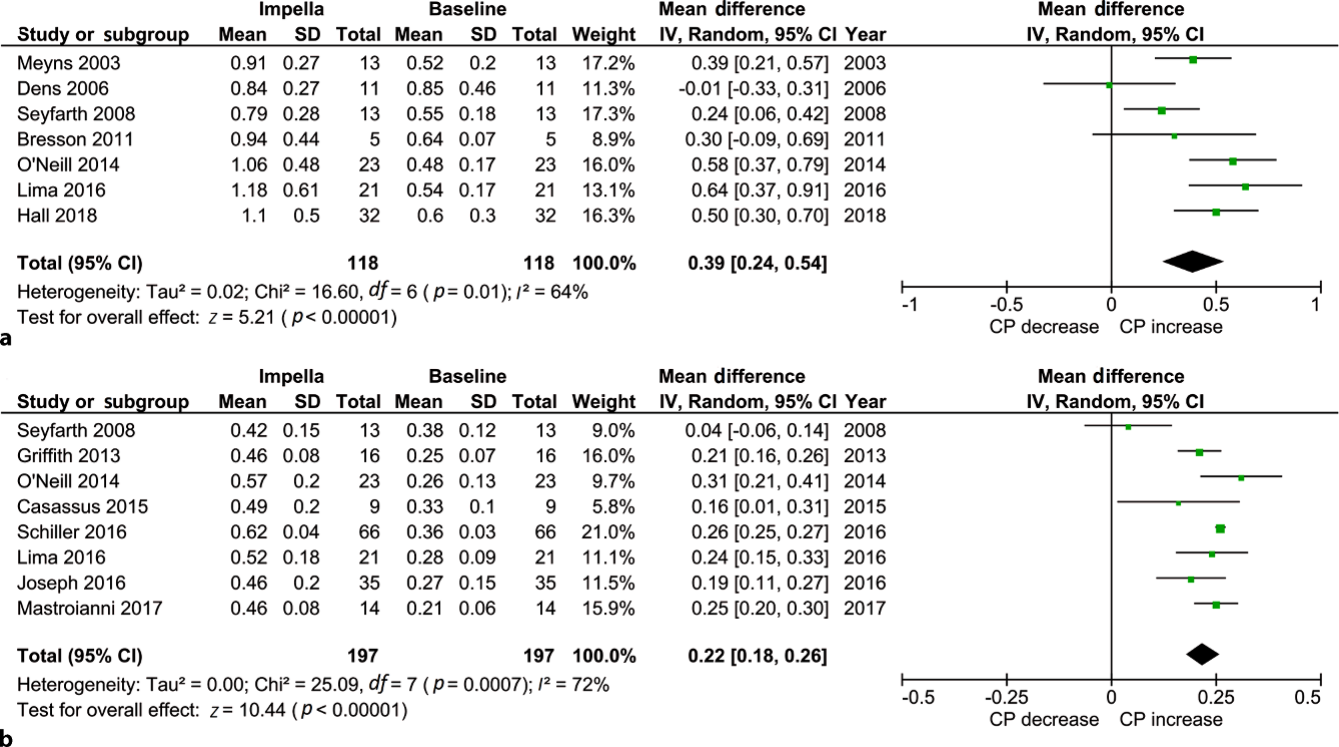
Forest plot of **a** cardiac power (*CP*) and **b** cardiac power index

CO and/or CI were reported in 258 (43%) patients (see Tab. 1). Using a random effect model, use of the Impella led to an increase in CP of 0.39 W [95% confidence interval (CI): 0.24, 0.54], (*p* = 0.01) and CPI 0.22 W/m^2^ (95% CI: 0.18, 0.26), (*p* < 0.01); see Tab. 2 and Fig. 2.

**Table 2:** Individual study results of haemodynamic support and survival

Study	Type of device	CP (W)		CPI (W/m^2^)		CO (l/min)		CI (l/min/m^2^)		MAP (mm Hg)		Survival (%)
		Baseline	Support	Baseline	Support	Baseline	Support	Baseline	Support	Baseline	Support	
Meyns [12]	5.0	0.52 ± 0.20	0.91 ± 0.27	–	–	4.1 ± 1.3	5.5 ± 1.3	–	–	57 ± 13	75 ± 13	46
Dens [13]	2.5	0.85 ± 0.46	0.84 ± 0.27	–	–	4.4 ± 1.9	4.8 ± 1.2	–	–	87 ± 25	79 ± 16	55
Seyfarth [14]	2.5	0.55 ± 0.18	0.79 ± 0.28	0.30 ± 0.12	0.42 ± 0.15	3.2 ± 0.8	4.1 ± 1.2	1.7 ± 0.5	2.2 ± 0.6	78 ± 16	87 ± 8	54
Bresson [15]	5.0	0.64 ± 0.07	0.94 ± 0.44	–	–	4 ± 0.55	5.9 ± 2.7	–	–	–	–	44
Griffith [16]	5.0	–	–	0.25 ± 0.07	0.46 ± 0.08	–	–	1.6 ± 0.4	2.5 ± 0.4	71 ± 13	83 ± 7.5	75
O’Neill [5]	2.5	0.48 ± 0.17	1.06 ± 0.48	0.26 ± 0.13	0.57 ± 0.20	3.4 ± 1.3	5.3 ± 1.7	1.9 ± 0.7	2.7 ± 0.7	63 ± 19	94 ± 23	51
Casassus [17]	2.5	–	–	0.33 ± 0.10	0.49 ± 0.20	–	–	2.2 ± 0.4	2.6 ± 0.7	67 ± 15	82 ± 13	59
Lima [18]	5.0	0.54 ± 0.17	1.18 ± 0.61	0.28 ± 0.09	0.52 ± 0.18	3.7 ± 1.3	5.8 ± 1.4	1.8 ± 0.5	2.9 ± 0.7	71 ± 11	82 ± 20	68
Schiller [19]	2.5/5.0	0.66 ± 0.2	–	0.36 ± 0.03	0.62 ± 0.04	–	–	2.2 ± 0.2	3.8 ± 0.2	73 ± 2	73 ± 2	58
Joseph [20]	2.5	–	–	0.27 ± 0.15	0.46 ± 0.20	3.5 ± 1.3	–	2.0 ± 0.6	2.4 ± 0.8	60 ± 28	87 ± 27	44
Mastroianni [21]	5.0	–	–	0.21 ± 0.06	0.46 ± 0.08	–	–	1.6 ± 0.4	2.8 ± 0.3	60 ± 9	74 ± 9	57
Hall [22]	5.0	0.6 ± 0.3	1.1 ± 0.5	–	–	3.7 ± 1.9	–	1.8 ± 0.6	2.8 ± 0.6	70 ± 11	–	67

Variables are presented as mean ± SD

*CO* cardiac output; *CI* cardiac index; *MAP* mean arterial pressure; *CP* cardiac power; *CPI* cardiac power index; *W* Watt; – not available

Use of the Impella 2.5 showed a mean pooled increase in CP and CPI of 0.29 W (95% CI: −0.02, 0.59), (*p* = 0.07), +48% and 0.18 W/m^2^ (95% CI: 0.06, 0.29), (*p* < 0.01), +58%, respectively. Use of the Impella 5.0 led to a mean pooled increase in CP and CPI of 0.46 W (95% CI: 0.35, 0.58), (*p* < 0.01), +82% and 0.27 W/m^2^ (95% CI: 0.17, 0.38), (*p* < 0.01), +102%. See Electronic Supplementary Material, online Figs. 1 and 2.

The majority of the patients received an Impella for CS after an AMI, which comprised 63% of the total study population. When analysing the AMI-CS specifically, the CPI increase was similar to that of the whole group (*n* = 258). See Electronic Supplementary Material, online Fig. 3.

**Fig. 3:**
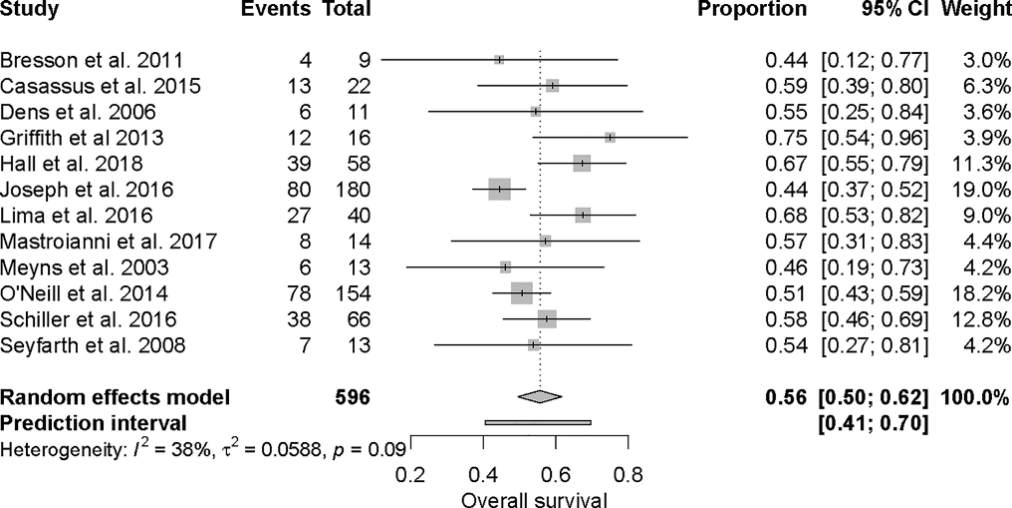
Forest plot of survival

**Fig. 4:**
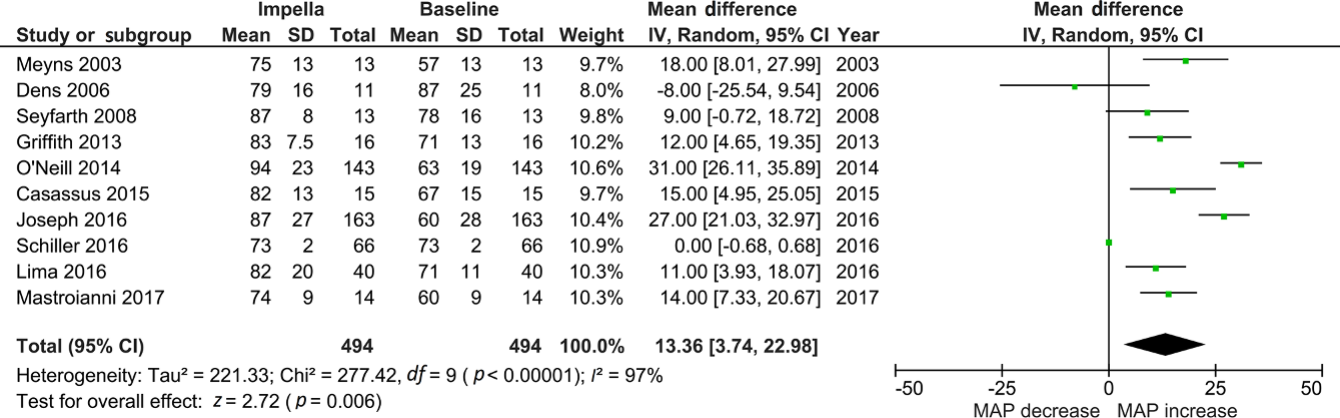
Forest plot of mean arterial pressure (*MAP*)

**Fig. 5:**
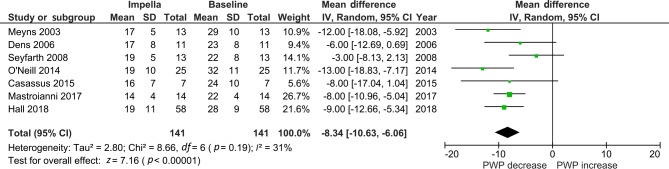
Forest plot of pulmonary wedge pressure (*PWP*)

The mean survival rate was 56% (95% CI: 0.50, 0.62), (*p* = 0.09), see Fig. 3. MAP increased with a pooled mean difference of 13 mm Hg (95% CI: 3.74, 22.98), (*p* < 0.01). PWP decreased when the Impella was used with a mean pooled difference of −8.30 mm Hg (95% CI: −10.63, −6.06), (*p* < 0.01). See Tab. 2, Figs. 4 and 5 and Electronic Supplementary Material.

#### Critical appraisal

**Fig. 6:**
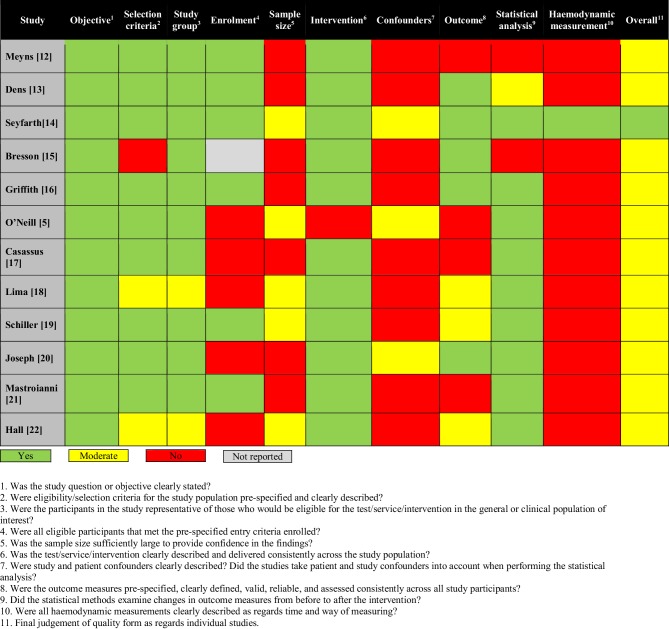
Adapted quality assessment for individual studies according to the U.S. Department of Health and Human Services. National Heart, Lung and Blood Institute

One study was considered of sufficiently good quality to show that Impella support results in increased CP and CPI [14]. Overall studies were considered to be of moderate quality, mostly due to lack of description of confounders and data acquisition protocol. On the other hand, all studies were comparable in terms of outcomes, study design, study population and type of support, which allowed us to conduct a meta-analysis (see Fig. 6).

## Discussion

To our knowledge, this is the first meta-analysis that has focused on the increase in CP and CPI during Impella support. This meta-analysis, including 258 patients from 12 studies, showed that the use of the Impella device significantly increases the CP by 0.39 W (+67%) and CPI by 0.22 W/m^2^ (+76%). When comparing the different Impella devices, the Impella 2.5 in general achieves a lower performance relative to the Impella 5.0 in both CP (+48% vs +82%) and CPI (+58% vs +102%).

The observed increase in CP(I) during Impella support, which has been shown to be a strong haemodynamic predictor of survival in CS [7, 8], should theoretically lead to a reduction in mortality. Extrapolating from the survival graph of Fincke et al., the increase of CP from 0.5 W to 0.9 W should decrease mortality from approximately 50% to 20% [7]. The overall percentage survival in our meta-analysis, 56%, is in line with two small randomised controlled trials [14, 23] and a propensity-matched analysis [24], which all compared the Impella CP to passive unloading with the intra-aortic balloon pump. This indicates that the relationship between (mechanical) haemodynamic improvement and survival is less evident than suggested.

In our study we focused mainly on CS after AMI (64% in our analysis). However, CS has a broad scope of aetiology. In some very specific indications, such as a biopsy-proven myocarditis, there is growing evidence of improved survival with Impella support [25]. In other indications for Impella support, such as the post-cardiotomy population (5% in our study), evidence is still limited and in need of further research. However, small registries show survival rates comparable with those of more invasive assist devices, such as surgically implantable ventricular assist devices [26]. Therefore, patient selection in terms of cause and reversibility of cause is an important determinant of survival in CS.

Within the CS-AMI group, patient selection might be an explanation for the lack of a clear survival benefit with improved haemodynamics. Patients with a relatively preserved cardiac function seem to have the best chance of survival and show a better haemodynamic improvement [27]. In patients who have no cardiac reserve, the intrinsic CP is unchanged and thus remains the Achilles’ heel of survival. Only when the affected myocardium is able to recover can the intrinsic CP increase and thereby improve outcome.

To achieve recovery of the myocardium Impella provides unloading, represented by a significant decrease of the PWP by 8 mm Hg in our meta-analysis. This clearly distinguishes mechanical support with the Impella device from medical therapy (inotropic or vasoactive agents), which increases the workload of the heart in order to improve the CPI [28]. Unloading of the left ventricle leads to reduced oxygen consumption and should thereby reduce infarct size in patients with CS-AMI. Animal studies have shown that unloading does reduce infarct size [1, 29], especially when support is started at an early stage. However, the clinical trial which investigated if unloading with Impella support would reduce the infarct size (MINI-AMI, Minimizing Infarct Size with Impella 2.5 Following PCI for Acute Myocardial Infarction, ClinicalTrials.gov identifier NCT01319760) was terminated due to a ‘change in company priority’. This raises questions as to whether the study was able to show positive results.

In terms of the timing of support, several studies suggest that early implantation of a mechanical assist device would improve survival [30–32]. Recent extensive animal studies showed that mechanical unloading of the left ventricle before coronary reperfusion limits the expression of proteolytic enzymes. This resulted in less cell decay, reduced infarction size and better haemodynamic performance [33]. A recent clinical trial also showed promising results when the Impella support was initiated before emergency PCI [34]. This is in contrast to our meta-analysis, in which support was given after almost 3 days after the onset of CS. The late initiation of support might preclude the potential benefit to survival rates. On the other hand, real-world data are refractory. In the 12-year experience of the Amsterdam Medical Centre there was no significant improvement of survival when support was initiated before PCI [35].

### Clinical and future perspectives

In the critical setting of CS, the Impella device improves the haemodynamic state and relieves congestion. However, in order to significantly improve outcomes, more research is needed. Patient selection and timing of Impella support may well be the crucial denominator that decides its effectiveness. To further optimise patient selection and to overcome heterogenic outcomes in future studies on MCS, we suggest a standard data set of core outcomes and measurements.

### Limitations

Eight of the 12 included studies were registries, which in general have a heterogeneous patient population, treatment and outcome. Additionally, 3 out of 12 studies are from the cVAD (catheter-based ventricular assist devices) registry, owned by Abiomed. Possible overlap cannot be excluded. When taking these studies out of the calculation, the overall results remain the same.

The key hindrance to providing an in-depth meta-regression analysis at the study level is the great disparity in the available data reported. Possible confounding factors are not always reported, including the use of vasoactive medication, clinical patient characteristics and the timing and completeness of measurements. Although the overall quality of the studies was considered moderate, all studies showed a uniform increase in CP(I). This was reflected by an acceptable heterogeneity score for the overall study group.

Furthermore, although the intrinsic CP(I) may be a strong predictor in CS-AMI, this relationship might be less strong for the CP(I) added by MCS. This distinction is crucial for adequate interpretation of our results. In addition, this meta-analysis focuses on the haemodynamic efficacy in the clinical setting, and merely reflects whether the pump is effective in increasing output. Procedure- and device-related complications (stroke, access bleeding, infection) are not included in this study, hampering the true reflection of clinical benefit for the patient.

### Conclusion

Our meta-analysis shows that short-term MCS with the Impella device is effective in increasing CP and CPI. Despite successfully increased CP with Impella support, the mortality seems to be in line with the survival rate without Impella use.

## Caption Electronic Supplementary Material

Supplemantery Fig. 1: Forest plot comparison of change in cardiac power (CP) between Impella 5.0 and 2.5.

Supplementary Fig. 2: Forest plot comparison of change in cardiac power index (CPI) between Impella 5.0 and impella 2.5.

Supplemetary Fig. 3: Forest plot of the cardiac power (index) (CP(I)) between cardiogenic shock patients based myocardial infarction (AMI-CS) and other cardiogenic shock patients (non AMI-CS).

Supplementary Table 1: PRISMA checklist used for this systematic review and meta-analysis.
