# Supplementary figures and images for: Haemodynamic efficacy of microaxial left ventricular assist device in cardiogenic shock: a systematic review and meta-analysis
Source: Neth Heart J. 2019 Dec 6;28(4):179–89. doi: 10.1007/s12471-019-01351-7 (PMC7113339; doi:10.1007/s12471-019-01351-7)

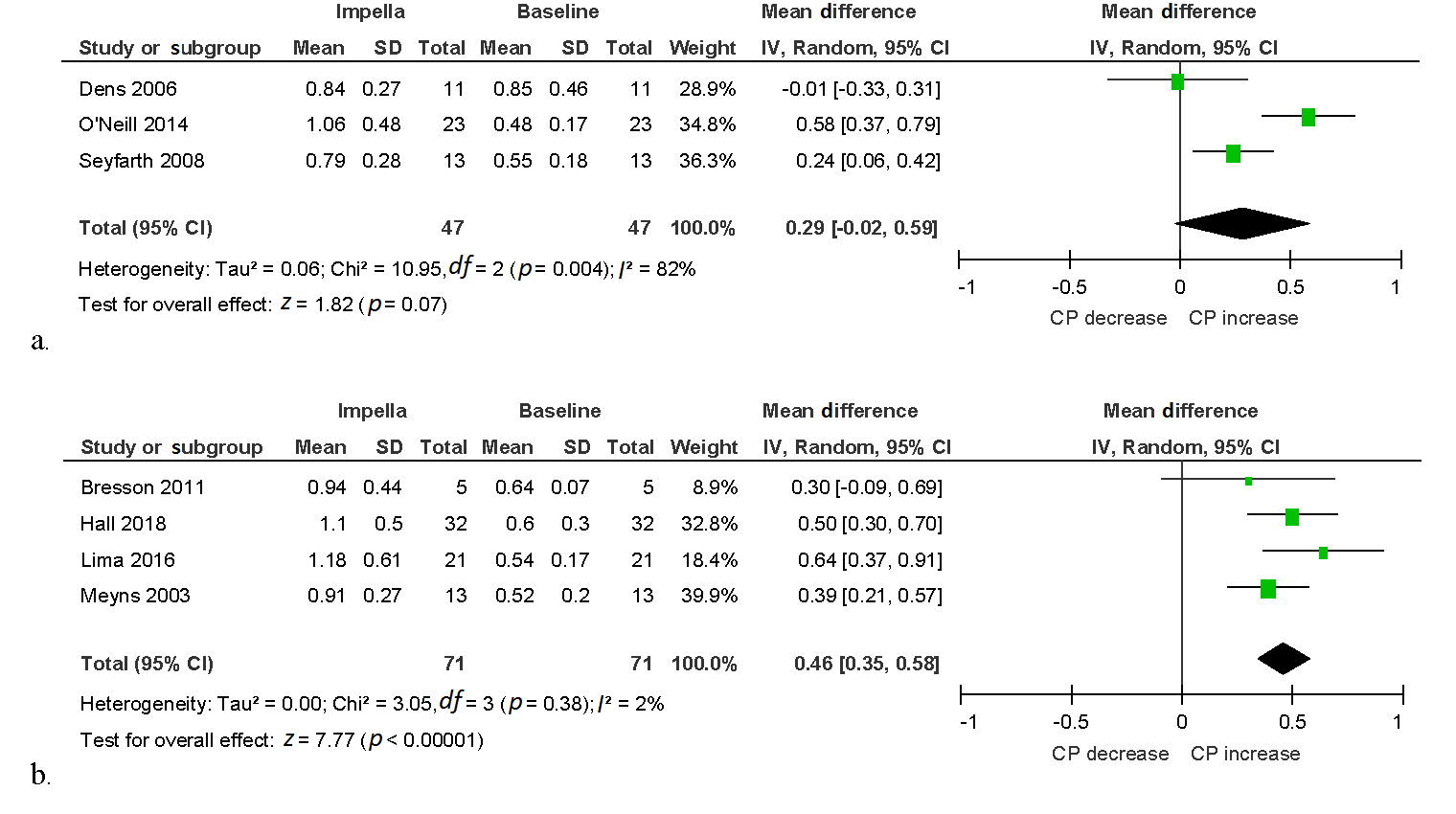

Supplement: Supplementary file 1 — Supplemantery Fig. 1: Forest plot comparison of change in cardiac power (CP) between Impella 5.0 and 2.5. [file 12471_2019_1351_MOESM1_ESM.tif]

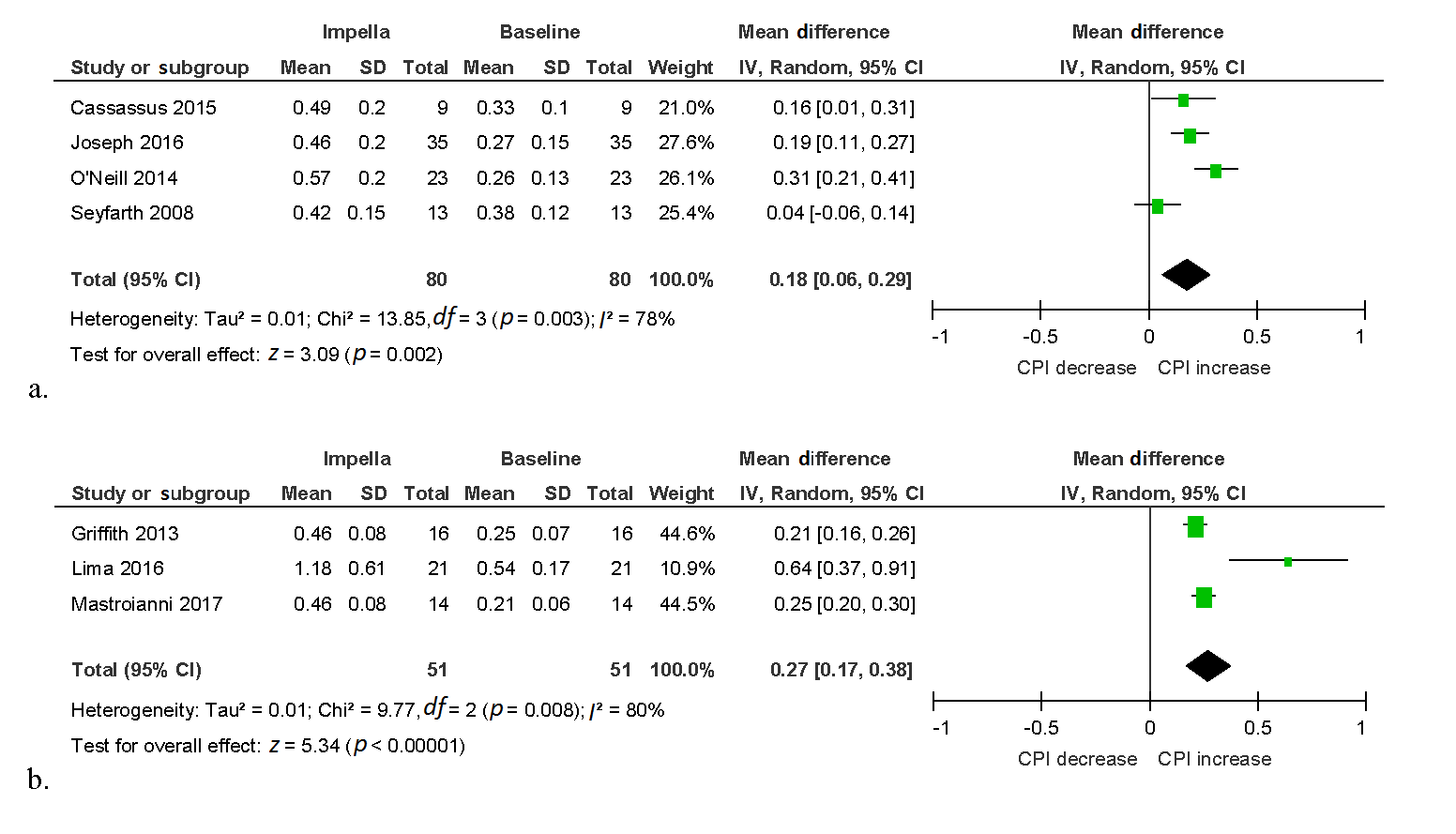

Supplement: Supplementary file 2 — Supplementary Fig. 2: Forest plot comparison of change in cardiac power index (CPI) between Impella 5.0 and impella 2.5. [file 12471_2019_1351_MOESM2_ESM.tif]

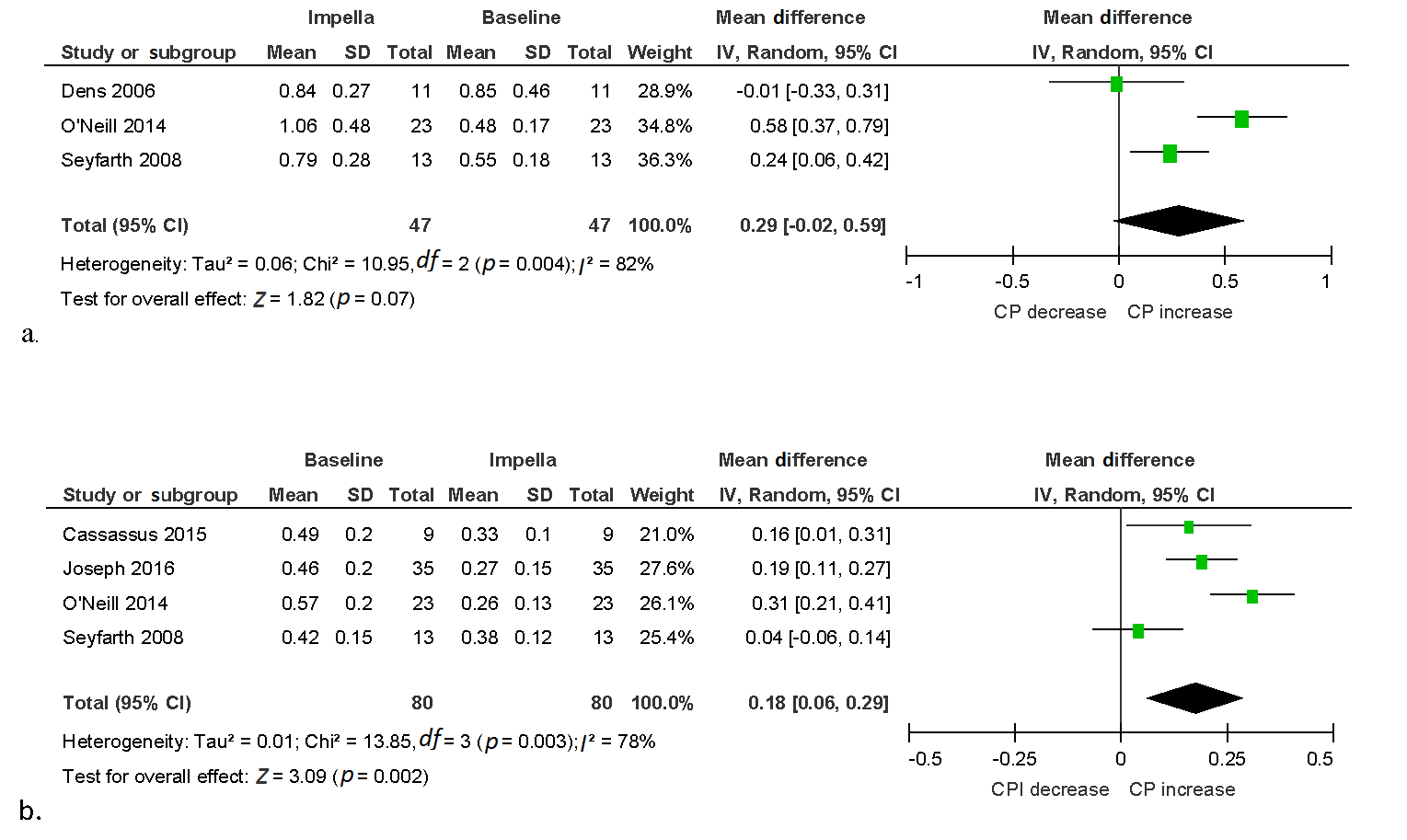

Supplement: Supplementary file 3 — Supplemetary Fig. 3: Forest plot of the cardiac power (index) (CP(I)) between cardiogenic shock patients based myocardial infarction (AMI-CS) and other cardiogenic shock patients (non AMI-CS). [file 12471_2019_1351_MOESM3_ESM.tif]
